# Dynamics of antibiotic resistance genes and the association with bacterial community during pig manure composting with chitin and glucosamine addition

**DOI:** 10.3389/fmicb.2024.1384577

**Published:** 2024-05-22

**Authors:** Bo Wang, Wenjie Chen, Chula Sa, Xin Gao, Su Chang, Yuquan Wei, Ji Li, Xiong Shi, Longli Zhang, Chunhua Zhang, Wenting Li, Haizhou Sun

**Affiliations:** ^1^Institute of Animal Nutrition and Feed, Inner Mongolia Academy of Agricultural and Animal Husbandry Sciences, Hohhot, China; ^2^Key Laboratory of Grass-Feeding Livestock Healthy Breeding and Livestock Product Quality Control (Co-construction by Ministry and Province), Ministry of Agriculture and Rural Affairs, Hohhot, China; ^3^Inner Mongolia Key Laboratory of Herbivore Nutrition Science, Hohhot, China; ^4^College of Resources and Environmental Science, Beijing Key Laboratory of Biodiversity and Organic Farming, China Agricultural University, Beijing, China; ^5^Yangtze Eco-Environment Engineering Research Center, China Three Gorges Corporation, Beijing, China; ^6^Beijing VOTO Biotech Co., Ltd, Beijing, China

**Keywords:** antibiotic resistance genes (ARGs), mobile genes elements (MGEs), bacterial communities, chitin, glucosamine, composting

## Abstract

In modern ecological systems, the overuse and misuse of antibiotics have escalated the prevalence of antibiotic resistance genes (ARGs) and mobile genetic elements (MGEs), positioning them as emerging environmental contaminants. Notably, composting serves as a sustainable method to recycle agricultural waste into nutrient-rich fertilizer while potentially reducing ARGs and MGEs. This study conducted a 47-day composting experiment using pig manure and corn straw, supplemented with chitin and N-Acetyl-D-glucosamine, to explore the impact of these additives on the dynamics of ARGs and MGEs, and to unravel the interplay between these genetic elements and microbial communities in pig manure composting. Results showed that adding 5% chitin into composting significantly postponed thermophilic phase, yet enhanced the removal efficiency of total ARGs and MGEs by over 20% compared to the control. Additionally, the addition of N-Acetyl-D-glucosamine significantly increased the abundance of tetracycline-resistant and sulfonamide-resistant genes, as well as MGEs. High-throughput sequencing revealed that N-Acetyl-D-glucosamine enhanced bacterial α-diversity, providing diverse hosts for ARGs and MGEs. Resistance mechanisms, predominantly efflux pumps and antibiotic deactivation, played a pivotal role in shaping the resistome of composting process. Co-occurrence network analysis identified the key bacterial phyla *Proteobacteria*, *Firmicutes*, *Gemmatimonadota*, and *Myxococcota* in ARGs and MGEs transformation and dissemination. Redundancy analysis indicated that physicochemical factors, particularly the carbon-to-nitrogen ratio emerged as critical variables influencing ARGs and MGEs. The findings lay a foundation for the developing microbial regulation method to reduce the risks of ARGs in animal manure composts.

## 1 Introduction

In the intricate realm of ecological systems, the rampant and improper use of antibiotics stands as a potent catalyst for the rapid surge of antibiotic resistance genes (ARGs) (Li et al., 2023). China ranks among the leading consumers of agricultural antibiotics worldwide, with its agricultural sector having consumed 30,903 tons of antibiotics in 2019 (Liu et al., 2023). Characterized by their slow metabolism and the capacity for horizontal gene transfer across bacterial communities, ARGs pervade the environment (Wang et al., 2015). The dangerous transmission of ARGs via horizontal gene transfer processes poses a latent threat to human and animals health, designating ARGs as emerging environmental contaminants (Zhang et al., 2020).

Composting, a sustainable method for converting residual organic material into nutrient-rich fertilizer, emerges as a promising avenue for recycling agricultural waste and enhancing soil quality (Wei et al., 2018; Zhang Y. et al., 2022; Zhang Z. et al., 2022). Given the distinct features of composting and the widespread use of antibiotics, composting plays a strategic role in managing antibiotic fermentation residues. The nuanced fluctuations in the abundance of ARGs, spanning sulfonamides to tetracyclines, within animal manure closely align with the behavioral nuances of microbial communities (Zou et al., 2020). Considering microbes are the primary carriers of ARGs, the shifting landscape of bacterial community structure during composting intricately shapes the abundance and dissemination of ARGs (Qiu et al., 2022). Moreover, mobile genetic elements (MGEs) play a crucial role in the horizontal transfer of ARGs within microbial communities, utilizing mechanisms like conjugation and transposon-plasmid interactions (Horne et al., 2023). The ebb and flow in ARG abundance harmonize significantly with antibiotic degradation dynamics and shifts in environmental factors (Han et al., 2022). Previous studies have demonstrated that elevated temperatures during aerobic composting can effectively reduce ARGs (Ahmed et al., 2023). However, the fundamental principles influencing the dynamics of ARGs abundance require further clarification. A comprehensive analysis of the response characteristics among microbial communities, physicochemical properties, and ARGs/MGEs during composting is crucial.

Chitin and N-Acetyl-D-glucosamine, a monomer of chitin, are intriguing components that can potentially influence the fate of ARGs and MGEs during composting. Chitin, a polysaccharide abundantly present in the exoskeletons of arthropods and fungi, is known for its ability to modulate microbial communities (Elieh-Ali-Komi and Hamblin, 2016), and is degraded by chitinase into chitosan and other derivatives under microbial action. These derivatives have been shown to possess highly effective antimicrobial properties. In particular, chitosan disrupts the integrity of bacterial cell membranes, leading to the leakage of cellular contents and ultimately resulting in cell death (Liu et al., 2004). As a substrate, chitin serves as a potential reservoir of carbon and nitrogen, influencing nutrient availability and shaping the microbial landscape during composting. The unique antibacterial activity of chitin derivatives may exert a pronounced inhibitory effect on the proliferation of microorganisms carrying ARGs and MGEs. Conversely, N-Acetyl-D-glucosamine may be a critical derivative influencing the fate of ARGs and MGEs in composting, contributing to the carbon and nitrogen pool, and potentially affecting microbial behavior through its derivatives (Bobbink et al., 2015). Investigating the impact of these components on the fate of ARGs and MGEs throughout the composting process is essential for understanding their multifaceted roles in shaping microbial communities and guiding the overall efficacy of composting as a sustainable strategy for managing antibiotic residues.

A 47-day composting experiment was conducted using pig manure and corn straw as raw materials, with chitin and N-Acetyl-D-glucosamine added as inhibitors to remove ARGs and MGEs from the compost. The objectives of this study were: (1) to investigate the impact of inhibitors on the abundance and removal efficiency of ARGs during composting; (2) to identify potential bacterial carriers of ARGs and MGE genes; and (3) to explore the key factors influencing the removal of ARGs and MGEs. The findings provide a foundation for developing microbial regulation methods to reduce the risks of ARGs in animal manure composts.

## 2 Materials and methods

### 2.1 Materials and experimental setup

Fresh pig manure was procured from the Changping Test Base at the Institute of Animal Sciences, Chinese Academy of Agricultural Sciences, located in Beijing, China. N-Acetyl-D-glucosamine and chitin were produced and supplied by Wuhan Lanabai Pharmaceutical Chemical Co., Ltd. Cornstalks were utilized to adjust the carbon-to-nitrogen ratio (C/N), sourced from Shangzhuang Experimental Station at China Agricultural University, Beijing, China. They underwent air-drying and were subsequent meticulously sieved through 2 mm sieves. Supplementary Table S1 displayed the basic properties of the raw materials for composting. Composting experiments were conducted in the reactors described by Chen et al. (2022). Pig manure was mixed with cornstalks in a ratio of 5:1 (wet weight basis). The initial C/N of the composting process was approximately 25, and the initial wet weight of each pile was 20 kg. Three groups were established: two experimental groups (PMNG and PMCH) and a control group (PM). In the PMNG group, materials were uniformly blended with 5% N-Acetyl-D-glucosamine, while in the PMCH group, materials were mixed with 5% chitin; the PM group did not receive any additional treatments. Each treatment had three replicates. The initial moisture content of the piles was adjusted to approximately 60%, and no additional water would be added throughout the composting process. Throughout composting, air was supplied from the bottom with an average airflow rate of approximately 0.2 L kg^−1^ min^−1^. To maintain a consistent oxygen supply, the piles were turned over at each sampling time. Sampling was conducted on days 0, 5, 12, 19, 26, 33, 40, and 47, with five subsamples collected from different locations within the pile profile and homogeneously mixed each time. Approximately 400 g of samples were collected from each pile. A portion of the samples was stored at 4°C for subsequent physical–chemical analyses, while the remaining samples were stored at −20°C for molecular extraction.

### 2.2 Physicochemical parameters analysis

Composting temperature was continuously monitored and recorded using a digital thermometer. Fresh samples were extracted with deionized water (DW) at a solid: liquid ratio of 1:10 (w/v, dry mass basis). Subsequently, pH and electrical conductivity (EC) were measured using a digital pH meter and an electrical conductivity meter (MP521, Sanxin, China). Total carbon and total nitrogen were quantified using a Vario MACRO cube elemental analyzer (Elementar Analysensystem, Germany), as described in Chen et al. (2023). The germination index (GI) was determined in accordance with the Chinese Standard for Organic Fertilizers (NY/T 525–2021). Total organic carbon (TOC) in the samples was analyzed using an organic carbon analyzer (TOC-Vcp, Shimadzu).

### 2.3 DNA extraction and high-throughput sequencing of bacterial 16S rRNA

Total genomic DNA was extracted using the FastDNA SPIN Kit for Soil (MP Biomedicals, Solon, OH, United States) following the manufacturer’s instructions. The purity and concentration of DNA for each sample were measured using a NanoDrop2000 (Thermo Fisher Scientific, Waltham, MA, United States). DNA integrity was verified through 1% agarose gel electrophoresis. The V3–V4 region of the 16S rRNA gene was amplified using primers 341F (F: 5′-CCTAYGGGRBGCASCAG-3′) and 806R (R: 5′-GGACTACNNGGGTATCTAAT-3′). Sequencing was performed on the Illumina MiSeq PE250 platform (Novogene Co., Ltd., Beijing, China). Raw 16S rRNA gene sequences were processed using QIIME2 (Quantitative Insights into Microbial Ecology, version 2022.2) as described by Bolyen et al. (2019). Reads were trimmed based on the Q30 minimum value and denoised using the Deblur method. All amplicon sequence variants were aligned with mafft, and a phylogenetic tree was constructed using fasttree2.

### 2.4 High-throughput sequencing of ARGs, MGEs, and 16S rRNA

Total genomic DNA for detecting ARGs, MGEs, and 16S rRNA was extracted using the FastDNA Spin Kit for Soil (MP Biomedicals) following the manufacturer’s instructions. Forty-nine ARGs for aminoglycoside (*Aac6-Aph2*, *aac(3)-xa*, *aac(6′)-Ib(aka aacA4)-01*, *aac(6′)-II*, *aac(6′)-Ib*, *aacA/aphD*, a*adD, aadE*, *aphA1*, *strB*), miltidrug (*acrA*, *acrB*, *arsA*, *mdtA*, *qnrD*, *qnrS2*), macrolide-lincosamide-streptogramin B (MLSB) (*ere(A)*, *erm(A)*, *lmrA*, *lnuA*, *mefA*, *mphA*, *msr(A)*, *vat(A)*, *vgaA*), sulfonamide (*dfrA1*, *folA*, *sul1*, *sul2*), tetracycline (*tetA*, *tetB*, *tetC*, *tetD*, *tetE*, *tetH*, *tetJ*, *tetK*, *tetM*, *tetO*, *tetR*, *tetS*, *tetT*, *tetW*, *tetX*), vancomycin (*vanA*, *vanB*, *vanC*) and trimethoprim (*dfrBmulti*, *dfrC*), eight MGEs, including integrase (*intl1*, *intl2*, *intl3*), plasmid (*tra-A*), Insertional_sequence (*IS3*, *IS26*, *ISCR1*) and transposase (*tnpA-1*) and one 16S rRNA were quantified using the WaferGen SmartChip Real-Time qPCR System (WaferGen Bio-systems, Fremont, CA, USA) with primers sourced from Anhui Microanaly Genetech Co., Ltd. The qPCR reactions were conducted in a 100 nL reaction system comprising 1x LightCycler 480 SYBR Green I Master Mix, 500 nM of each primer, and 2 ng μL^−1^ DNA template. The reaction mixtures were loaded into a SmartChip Cycler (WaferGen Biosystems) using a multi-sample dispenser (WaferGen Biosystems) following the 144 (assays) × 36 (samples) model. Each sample was measured in triplicate. The reaction protocol involved pre-denaturation at 95°C for 10 min, denaturation at 95°C for 30 s, and annealing at 60°C for 30 s. The qPCR results were automatically analyzed using qPCR software, with CT = 31 set as the detection threshold. No detections in any of the three replicates were considered negative results.

The gene copy numbers of ARGs, MGEs, and 16S rRNA were determined using the formula: gene copy number = 10^(31 − CT) × (10/3)^. The relative abundance of ARGs or MGEs was calculated as the ratio of the gene copy number of ARGs or MGEs to the gene copy number of 16S rRNA. Quantification of the 16S rRNA genes was performed using a QuantStudio 6 Flex Real-Time PCR analyzer (Applied Biosystems, Waltham, MA, USA). The qPCR reaction system for 16S rRNA genes consisted of 10 μL, comprising 5 μL 2x Mix, 0.5 μL forward primer, 0.5 μL reverse primer, 0.2 μL 5x ROX dye, 2.8 μL nucleotide-free water, and 1 μL DNA template. The reaction conditions involved enzyme activation at 95°C for 10 min, followed by 40 cycles of denaturation at 95°C for 15 s, and annealing at 60°C for 1 min. Each PCR reaction was performed in triplicate for technical consistency (Chen et al., 2024).

### 2.5 Statistical analysis

The physicochemical data were visualized using Origin Pro 2022. Heatmaps were generated in R Version 4.1.3 using the pheatmap package. Alpha diversity estimates, including Shannon diversity, Pielou’s evenness, and Chao1 richness, were calculated using the Vegan package. Co-occurrence network analysis between bacteria and ARGs/MGEs was conducted using the psych package with the Spearman correlation coefficient (*r* ≥ 0.96, *p* ≤ 0.01) and visualized using Gephi 0.9.6. Redundancy Analysis (RDA) was performed using Canoco 5 for Windows to identify the key physicochemical factors influencing the variations in ARGs.

## 3 Results

### 3.1 Changes of monitoring physicochemical parameters

The temperature was consistently maintained above 50°C for more than 6 days in all three treatments (Supplementary Figure S1), effectively eliminating the majority of pathogens and weed seeds. The temperature changes in the PM and PMNG treatments exhibited similar patterns, while in the PMCH treatment, a distinct lagged thermophilic phase was observed. With the progression of the composting process, the gradual decomposition of chitin facilitated the transition of PMCH into the thermophilic phase. After day 38, all piles reached the maturation phase, and the temperatures stabilized. On the 12th day of composting, the pH value in the PMCH treatment was 7.0, significantly lower than the other two treatments. As composting progressed, the pH values in all treatments increased to a range of 7.8–8.3 (Supplementary Figure S1). The EC values in the PM and PMNG treatments showed a declining trend, whereas in the PMCH treatment, the EC value initially increased and then decreased. By the end of composting, the EC values in all treatments were below 4 mS/cm (Supplementary Figure S1). The C/N ratios in PM, PMNG, and PMCH decreased, respectively, to 13.4, 11.2, and 13.3 at the end of composting. The GI for all three piles exceeded 90%, indicating the maturation of the composting process (Supplementary Figure S1). Composting is a process of organic mineralization and humification, and the TOC content in all three treatments exhibited a noticeable decrease during composting (Supplementary Figure S1), suggesting the microbial mineralization and transformation of organic matter.

### 3.2 Variation of ARGs and MGEs

**Figure 1:**
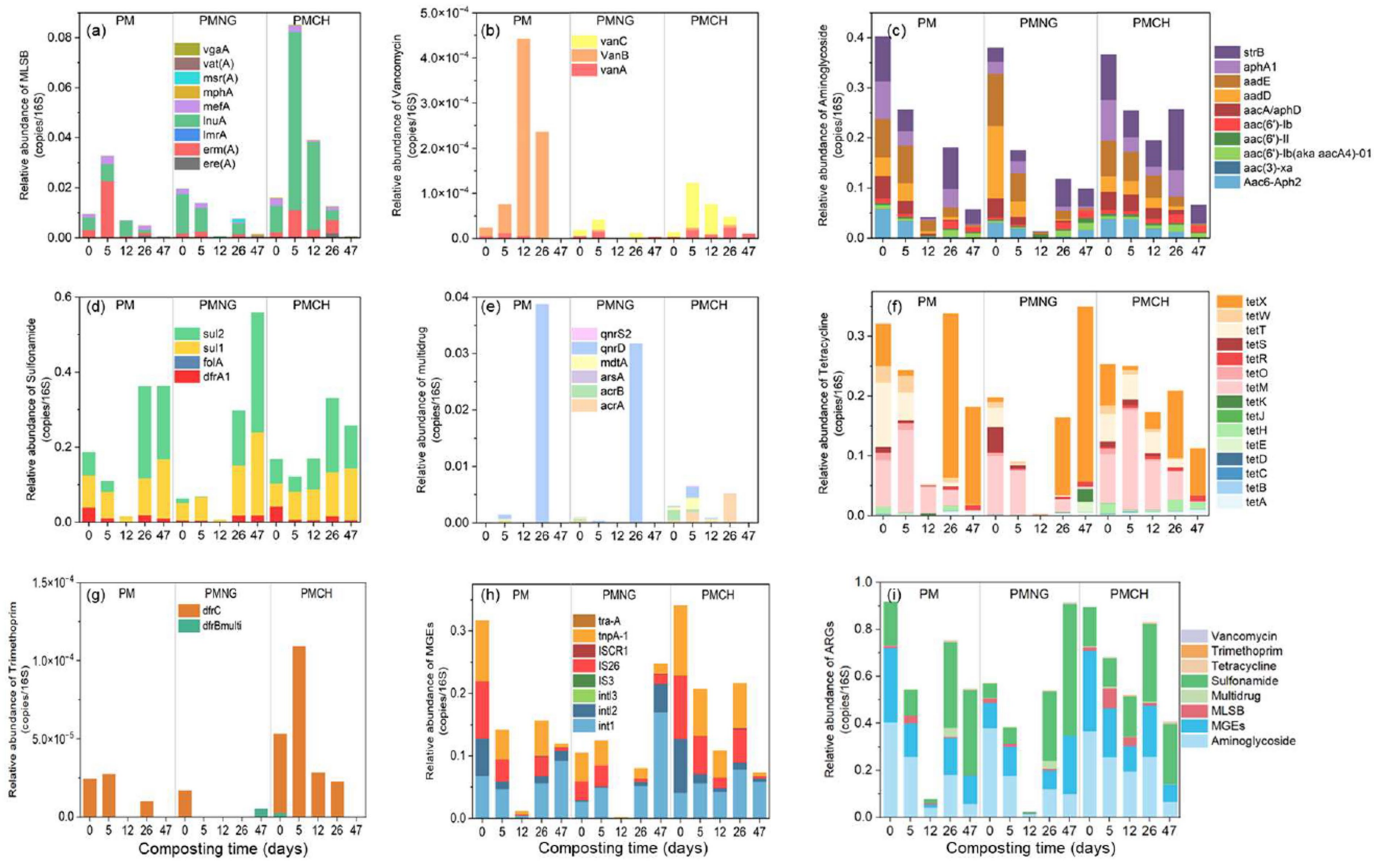
Variations of relative abundance of antibiotic resistance genes (ARGs) and mobile genetic elements (MGEs) in different treatments during composting. The relative abundance dynamics of gene subtypes in three treatments [**A**, Macrolide-lincosamide-streptogramin B (MLSB); **B**, Vancomycin; **C**, Aminoglycosides; **D**, Sulfonamide; **E**, multidrug; **F**, Tetracycline; **G**, Trimethoprim; **H**, MGEs]. The relative abundance dynamics of ARGs and MGEs at the level of antibiotic classification in three treatments **(I)**.

**Figure 2:**
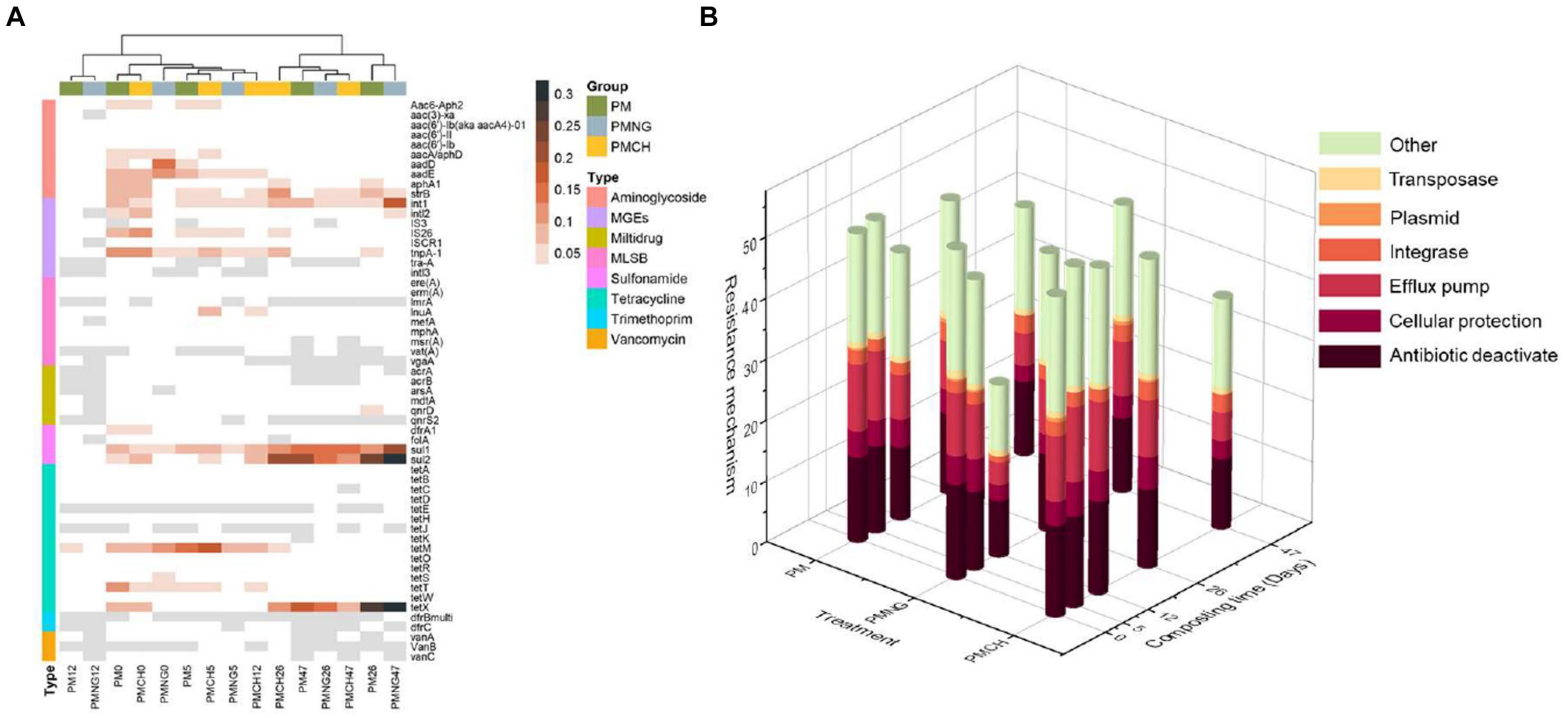
**(A)** Heatmaps showing the relative abundance (copies per 16S rRNA gene) of gene subtypes in different treatments during composting. **(B)** Relative abundance of ARGs according to resistance mechanism in different treatments during composting.

In this study, 57 common ARGs and MGEs subtypes were quantified, encompassing resistance to aminoglycoside (10), multidrug (6), MLSB (9), sulfonamide (4), tetracycline (15), vancomycin (3), trimethoprim (2) and MGEs (8) (Figure 1). The resistance mechanisms of ARGs were assessed. Efflux pumps and antibiotic deactivation were identified as dominant mechanisms in all samples, both in terms of detected gene numbers and gene abundances (Figure 2).

At the initial stage of composting, the relative abundance of total ARGs and MGEs per 16S rRNA in PM, PMNG, and PMCH ranged from 4.7 × 10^−1^ to 6.0 × 10^−1^ and 1.1 × 10^−1^ to 3.4 × 10^−1^, respectively (Figure 1I). Throughout the composting process, substantial differences were observed among the three treatments in the dynamics of ARGs and MGEs (Figure 1I). Specifically, in the PM and PMNG treatments, the total abundance of ARGs and MGEs reached its minimum on day 12, while in the PMCH treatment, the minimum abundance occurred on day 47 (Figure 1I). During the thermophilic phase (from day 0 to day 12), the removal efficiencies of total ARGs and MGEs in PM, PMNG, and PMCH were 91.4% for PM, 96.0% for PMNG, and 41.9% for PMCH; subsequently, the efficiencies were 96.2, 98.1, and 68.1%, respectively (Supplementary Figure S2). At the same time, the removal efficiency of ARGs and MGEs in the PMCH treatment was significantly lower than in the PM and PMNG treatments. The delayed onset of the thermophilic phase in PMCH resulted in lower removal rates of total ARGs and MGEs on the twelfth day. Additionally, the PM treatment exhibited varying degrees of enrichment for vancomycin-resistant genes, while the PMCH treatment showed enrichment for vancomycin, tetracycline, and MLSB resistance genes (Supplementary Figure S2). After 26 days of composting in the cooling phase, the removal rates of total ARGs and MGEs in PM, PMNG, and PMCH decreased to 17.7, 5.3, 7.3, and 50.7%, 23.7, 36.5%, respectively (Supplementary Figure S2). Simultaneously, all three treatments exhibited enrichment for tetracycline, sulfonamide, and multidrug resistance genes, while PM and PMCH treatments continued to show enrichment for vancomycin-resistant genes. At the end of composting, the removal rates of total ARGs in PM, PMNG, and PMCH were 40.2, −59.9%, and 54.5%, respectively (Supplementary Figure S2). For MGEs, the removal rates in PM, PMNG, and PMCH were 62.1, −135.4%, and 78.5%, respectively. The addition of chitin significantly enhanced the removal rates of total ARGs and MGEs in composting. It is noteworthy that PMNG significantly enriched ARGs and MGEs, primarily including tetracycline (*tetA*, *tetD*, *tetE*, *tetK*, *tetR*, *tetX*), sulfonamide (*dfrA1*, *sul1*, *sul2*), and MGEs (*intl1*, *intl2*, *intl3*, *IS3*, *ISCR1*) (Figures 1, 2). PM and PMCH also exhibited varying degrees of enrichment for tetracycline (*tetA*, *tetD*, *tetR*, *tetX*) and sulfonamide (*sul1*, *sul2*) (Figures 1, 2).

### 3.3 Changes in bacterial communities

**Figure 3:**
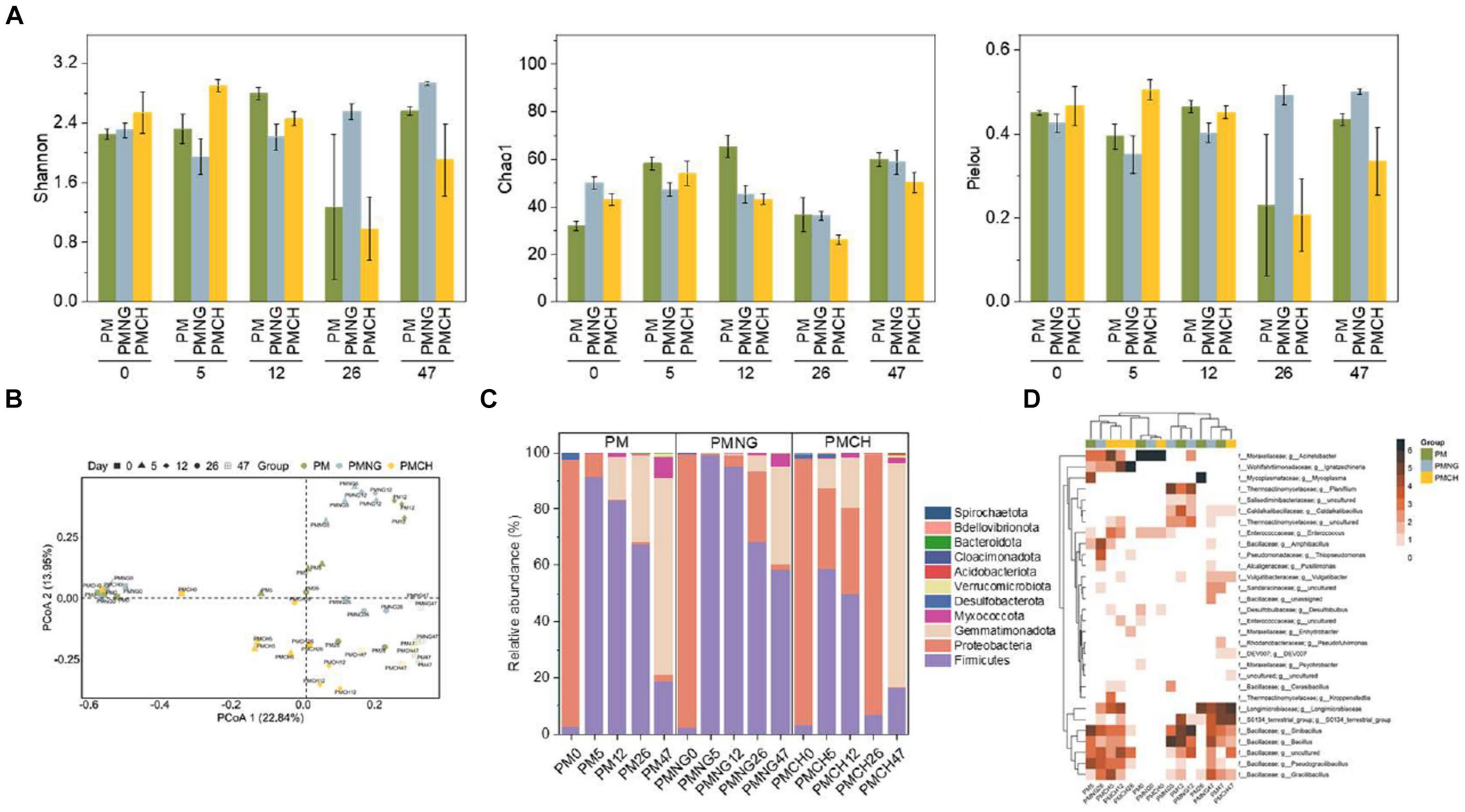
**(A)** Alpha diversity indices including Shannon diversity, Chao1 richness, and Pielou’s evenness of bacterial communities during composting in different treatments. **(B)** The principal coordinates analysis (PCoA) of bacterial communities in different treatments. **(C)** The relative abundance of the main phyla. **(D)** the relative abundance of top genera.

Shannon diversity, Pielou’s evenness, and Chao1 richness were employed to characterize the α-diversity of bacterial communities. At the end of composting, the PMNG treatment exhibited significantly higher Shannon diversity and Pielou’s evenness compared to the other two treatments, indicating that the addition of N-Acetyl-D-glucosamine significantly enhances the α-diversity of bacteria, providing a more diverse bacterial hosts for ARGs and MGEs, leading to gene enrichment (Figure 3A). Moreover, the dynamics of bacterial communities exhibited consistency with changes in ARGs and MGEs (Figure 3), suggesting a strong positive correlation between bacterial communities and ARGs/MGEs. Principal Coordinate Analysis (PCoA) based on the Bray-Curtis distance matrix revealed that bacterial communities in all samples during composting could be categorized into three groups (Figure 3B). However, on day 47, the bacterial communities in PM and PMCH tended to be similar. This result could explain the trends in the changes of ARGs and MGEs (Figure 1). At the phylum level, significant differences in bacterial composition were observed among PM, PMNG, and PMCH during the composting process (Figure 3C). *Firmicutes*, *Proteobacteria*, *Gemmatimonadota*, and *Myxococcota* were predominant phyla shared by all three treatments, constituting over 95% of all phyla. At the end of composting, the abundance of *Firmicutes* in PMNG was significantly higher than in the other two treatments. This provides a reasonable explanation for the enrichment of ARGs in the PMNG treatment and the eventual rebound in ARGs removal rates in the other treatments. The hierarchical clustering heatmap illustrates the dynamics of the top 30 bacterial communities at the genus level, standardized using the log2 function (Figure 3D). Bacterial communities across all treatments clustered into three groups, with higher abundances in the group comprising *Longimicrobiaceae* (*Gemmatimonadota*), *S0134_terrestrial_group* (*Gemmatimonadota*), *Sinibacillus* (*Firmicutes*), *Bacillus* (*Firmicutes*), *Pseudogracilibacillus* (*Firmicutes*), and *Gracilibacillus* (*Firmicutes*). It can be inferred that on the 12th day of composting, the high abundance of *Bacillus* (3.4–30.0%) across all treatments contributed to a significant reduction in the levels of ARGs and MGEs in composting (Figure 3D).

### 3.4 Relationships between bacterial communities, ARGs, and MGEs during composting

**Figure 4:**
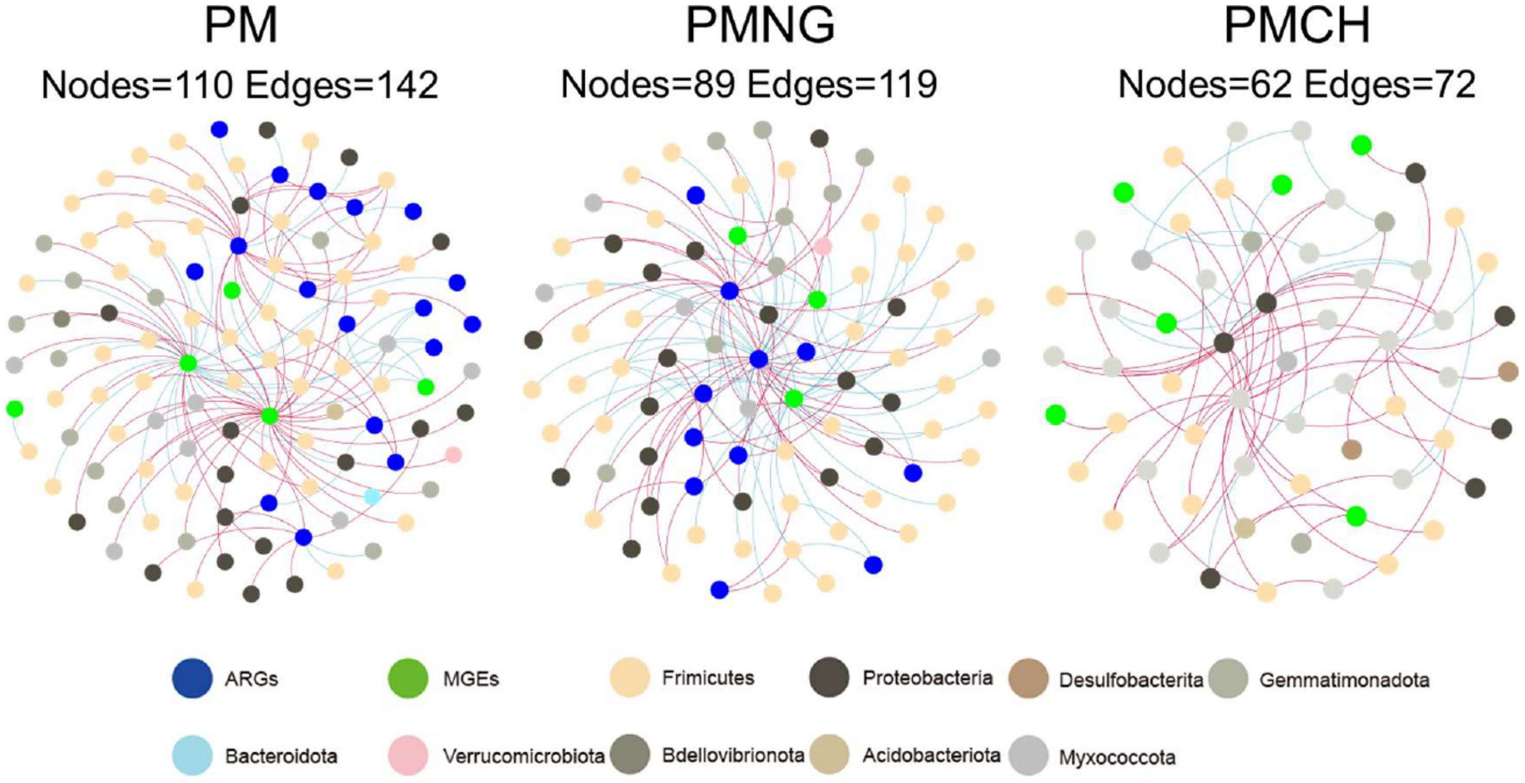
Co-occurrence network analysis showing the associations between ARGs/MGEs (based on relative abundance) and bacterial community (based on OTUs) in different treatments during composting.

The co-occurrence networks among ARGs, MGEs, and microbial communities (OTUs) for the three treatments are depicted in Figure 4. The network exhibits nodes (110, 89, and 62) and edges (142, 119, and 72) in PM, PMNG, and PMCH, respectively (Supplementary Table S2). Across PM (8), PMNG (5), and PMCH (6) bacterial phyla, the network identifies potential hosts for 22 ARGs and 8 MGEs, suggesting their crucial interactions. The predominant bacterial phyla associated with ARGs and MGEs are *Proteobacteria*, *Firmicutes*, *Gemmatimonadota*, and *Myxococcota*, highlighting their significance in the transformation and dissemination of ARGs and MGEs. Network analysis also allows for the identification of the most prevalent genes in different treatments. Noteworthy ARGs across all treatments include *vanA*, *IS3*, and *intl3* in PM; *acrB*, *arsA*, and *tra-A* in PMNG; and *vat(A)* and *qnrS2* in PMCH. *IS3* is identified as the most abundant host across all three treatments (Figure 4). Therefore, specific attention should be directed toward subtypes *vanA, IS3, intl3, acrB, arsA, tra-A, vat(A)*, and *qnrS2* during the composting of pig manure.

**Figure 5:**
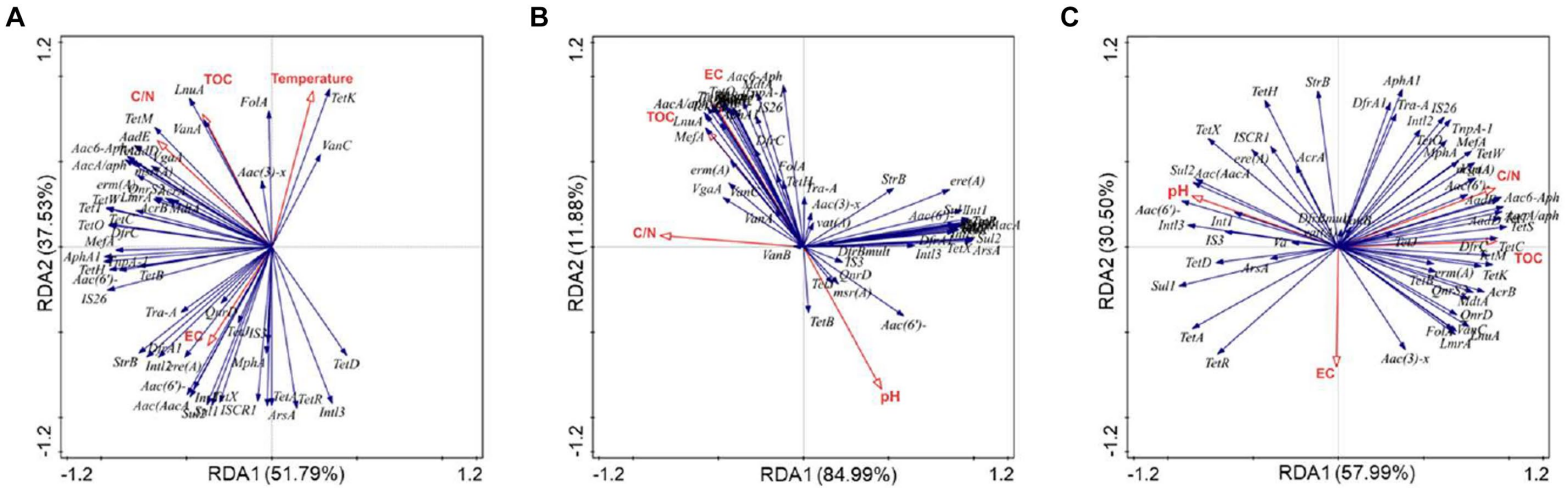
Redundancy analysis based on bacterial communities, environmental factors, ARGs, and MGEs during PM **(A)**, PMNG **(B)**, PMCH **(C)** composting.

Based on redundancy analysis, we investigated the relationships among physicochemical factors, MGEs, and ARGs (Figure 5). The analysis revealed that the carbon-to-nitrogen ratio (C/N) emerged as a crucial factor influencing ARGs and MGEs across the three treatments (*p* < 0.05). In other words, C/N ratio was identified as a key variable shaping antibiotic resistance during the composting process.

## 4 Discussion

The findings from this study elucidated the complex dynamics of ARGs and MGEs during the composting process and their interactions with microbial communities. Notably, the antimicrobial properties of chitin significantly influenced the fate of ARGs and MGEs. The lagged thermophilic phase in the PMCH treatment demonstrated that chitin’s inhibitory effects on microbial activity could be attributed to its degradation into antimicrobial derivatives such as chitosan, which disrupt bacterial cell membranes and hinder the proliferation of ARG-carrying microorganisms (Aranaz et al., 2009; Benhabiles et al., 2012).

The observed variations in ARG and MGE abundance across different composting phases suggested that both biotic and abiotic factors significantly influenced gene transfer and resistance mechanisms. The early reduction in ARGs and MGEs during the thermophilic phase aligned with previous findings that high temperatures can reduce the viability of ARG-carrying bacteria (Liao et al., 2019). However, the rebound in ARGs and MGEs levels during the cooling phase underscored the resilience of certain bacterial populations and the complexity of ARG dynamics, which were not solely dependent on temperature but also on microbial community structure (Qiu et al., 2021).

The differential effects of N-Acetyl-D-glucosamine addition highlighted its potential to alter bacterial community structure and function. The significant increase in α-diversity within the PMNG treatment suggested that N-Acetyl-D-glucosamine might enhance microbial heterogeneity, potentially providing a wider range of bacterial hosts for ARGs and thus influencing gene enrichment patterns (Liu et al., 2021; Zhu et al., 2023). This was corroborated by the strong positive correlation observed between bacterial community dynamics and ARG/MGE patterns, emphasizing the critical role of microbial ecology in ARG propagation.

Furthermore, network analysis revealed crucial interactions between bacterial phyla and ARG/MGEs, highlighting the roles of *Proteobacteria*, *Firmicutes, Gemmatimonadota*, and *Myxococcota* in the ecological networks that facilitate ARG and MGE dissemination (Yue et al., 2021). It has been reported that the phyla Firmicutes and Proteobacteria may be primarily responsible for carrying and disseminating multiple ARGs, as some members of the Actinobacteria and Firmicutes are producers of antibiotics, and previous studies have also reported that most antibiotic-resistant bacteria belong to Gram-positive bacteria (Mindlin et al., 2008; Allen et al., 2010; Qiu et al., 2021). The identified associations between these phyla and ARG/MGE abundance underscored the need for targeted interventions that consider the microbial carriers of resistance genes.

Among factors influencing composting efficiency and microbial communities, the C/N ratio has been recognized as a pivotal determinant (Zhu et al., 2021). Carbon and nitrogen are essential nutrients for microbial metabolism, and their concentrations dictate the enrichment levels of composting microorganisms with distinct nutritional preferences. This, in turn, influences the potential hosts of ARGs and MGEs. Wei et al. (2020) reported that composting with an initially higher C/N ratio (30.1) exhibited greater efficacy in ARG removal compared to composting with lower initial C/N ratios (20.1 or 25.1).

However, the study was not without its limitations. The study focused on a limited number of ARGs and MGEs, which, although representative, do not encompass the entire spectrum of antibiotic resistance genes that might be present in animal manure. This limitation restricts the breadth of our conclusions and suggests the need for broader surveillance of ARGs and MGEs to fully understand their dynamics during composting. Furthermore, while the study highlighted the potential of chitin and N-Acetyl-D-glucosamine to influence the fate of ARGs and MGEs, the mechanisms by which these additives impact microbial communities and gene transfer are not fully understood. More detailed mechanistic studies are required to elucidate how these substances interact at the molecular level with microbial populations and environmental factors in the compost matrix.

Looking forward, it was essential to develop a more comprehensive understanding of how different composting amendments interact not just with microbial communities but also with the physicochemical parameters of the composting environment. Experimental designs might also include a wider array of ARGs and MGEs, particularly those emerging in resistance profiles globally, to ensure that the strategies developed are robust and effective against a broad spectrum of threats (Zhang Y. et al., 2022; Zhang Z. et al., 2022). Additionally, the introduction of metagenomic sequencing could provide deeper insights into the microbial pathways and genetic transfers occurring during composting, thus refining our approaches to managing ARGs in these systems (Werner et al., 2022).

## 5 Conclusion

This study revealed the effect of chitin and glucosamine addition on the dynamics of ARGs and MGEs during pig manure composting, along with the associated microbial community dynamics. The addition of chitin or N-Acetyl-D-glucosamine, significantly influenced the removal rates and enrichment of ARGs and MGEs, with the removal rate of total ARGs and MGEs in PMCH treatment being over 20% higher than in the PM treatment. The assessment of resistance mechanisms identified efflux pumps and antibiotic deactivation as dominant mechanisms, highlighting their roles in shaping the resistome of composting piles. The addition of N-Acetyl-D-glucosamine enhanced the α-diversity of bacteria and provided diverse bacterial communities as potential hosts for ARGs and MGEs. *Proteobacteria*, *Firmicutes*, *Gemmatimonadota*, and *Myxococcota* were crucial players in the transformation and dissemination of ARGs and MGEs. The physicochemical factor, particularly the C/N, emerged as a critical variable influencing ARGs and MGEs during composting.

## Data availability statement

High-throughput sequencing for the purified 16S rRNA gene fragments were performed using the Illumina sequencing platform of Hiseq2500 by Novogene (Beijing, China). The sequences were submitted to the NCBI repository, accession number PRJNA1110395.

## Author contributions

BW: Resources, Validation, Writing – original draft. WC: Conceptualization, Data curation, Methodology, Visualization, Writing – original draft. CS: Software, Writing – original draft. XG: Formal analysis, Writing – original draft. SC: Visualization, Writing – original draft. YW: Conceptualization, Funding acquisition, Project administration, Resources, Supervision, Writing – original draft, Writing – review & editing. JL: Funding acquisition, Resources, Writing – review & editing. XS: Formal analysis, Resources, Writing – review & editing. LZ: Investigation, Writing – review & editing. CZ: Data curation, Writing – original draft. WL: Methodology, Writing – original draft. HS: Project administration, Resources, Writing – review & editing.
