# Supplementary material for: Dynamics of antibiotic resistance genes and the association with bacterial community during pig manure composting with chitin and glucosamine addition
Source: Front Microbiol. 2024 May 22;15:1384577. doi: 10.3389/fmicb.2024.1384577 (PMC11150687; doi:10.3389/fmicb.2024.1384577)
Supplement: Supplementary file 1 [file Data_Sheet_1.DOCX]

**Dynamics of antibiotic resistance genes and the association with bacterial community during pig manure composting with** **chitin and** **glucosamine addition**

Bo Wang^1,2,3,#,*^, Wenjie Chen^4,#^, Chula Sa^1,2,3^, Xin Gao^4^, Su Chang^4^, Yuquan Wei^4,*^, Ji Li^4^, Xiong Shi^5^, Longli Zhang^6^, Chunhua Zhang^1,2,3^, Wenting Li^1,2,3^, Haizhou Sun^1,2,3,*^

^1^ Institute of Animal Nutrition and Feed, Inner Mongolia Academy of Agricultural & Animal Husbandry Sciences, Hohhot, 010031, China

^2^ Key Laboratory of Grass-Feeding Livestock Healthy Breeding and Livestock Product Quality Control (Co-construction by Ministry and Province), Ministry of Agriculture and Rural Affairs, Hohhot, 010031, China

^3^ Inner Mongolia Key Laboratory of Herbivore Nutrition Science, Hohhot, 010031, China

^4^ College of Resources and Environmental Science, Beijing Key Laboratory of Biodiversity and Organic Farming, China Agricultural University, 100193, Beijing, China

^5^ Yangtze Eco-Environment Engineering Research Center, China Three Gorges Corporation, Beijing 100038, China

^6^ Beijing VOTO Biotech Co., Ltd, 100193 Beijing, China

# Authors contributed equally to this work

* Corresponding author

E-mail: wangbo595@126.com (B. Wang); weiyq2013@gmail.com (Y. Wei); sunhaizhou@china.com (H. Sun)

Supporting Information Includes:

- 2 tables

- 2 figures

**Table S1** Basic characteristics of composting raw materials.

| Raw material | Moisture content  (%) | pH | EC (mS·cm^-1^) | C/N | TOC (g/kg) | GI  (%) |
| --- | --- | --- | --- | --- | --- | --- |
| Pig manure | 73.09±0.80 | 7.68±0.12 | 3.45±0.21 | 15.77±0.30 | 1.62±0.00 | - |
| Cornstalks | 8.16±0.27 | 7.33±0.04 | 2.57±0.19 | 36.32±3.58 | 1.46±0.06 | 0.76±0.10 |

Values in parentheses are standard deviations of mean values (n = 3).

C/N, the ratio of total carbon to total nitrogen; TOC, total organic carbon; GI, germination index.

**Table S2** Co-occurrence network nodes information.

| Group | Node name | Domain | Phylum | Class | Order | Family | Genus |
| --- | --- | --- | --- | --- | --- | --- | --- |
| PM | vgaA | MLSB | ARGs | ARGs | ARGs | ARGs | ARGs |
| PM | vanC | Vancomycin | ARGs | ARGs | ARGs | ARGs | ARGs |
| PM | vanA | Vancomycin | ARGs | ARGs | ARGs | ARGs | ARGs |
| PM | tnpA-1 | MGEs | MGEs | MGEs | MGEs | MGEs | MGEs |
| PM | tetO | Tetracycline | ARGs | ARGs | ARGs | ARGs | ARGs |
| PM | tetH | Tetracycline | ARGs | ARGs | ARGs | ARGs | ARGs |
| PM | tetB | Tetracycline | ARGs | ARGs | ARGs | ARGs | ARGs |
| PM | strB | Aminoglycoside | ARGs | ARGs | ARGs | ARGs | ARGs |
| PM | msr(A) | MLSB | ARGs | ARGs | ARGs | ARGs | ARGs |
| PM | mphA | MLSB | ARGs | ARGs | ARGs | ARGs | ARGs |
| PM | IS3 | MGEs | MGEs | MGEs | MGEs | MGEs | MGEs |
| PM | IS26 | MGEs | MGEs | MGEs | MGEs | MGEs | MGEs |
| PM | intl3 | MGEs | MGEs | MGEs | MGEs | MGEs | MGEs |
| PM | int1 | MGEs | MGEs | MGEs | MGEs | MGEs | MGEs |
| PM | dfrC | Trimethoprim | ARGs | ARGs | ARGs | ARGs | ARGs |
| PM | dfrA1 | Sulfonamide | ARGs | ARGs | ARGs | ARGs | ARGs |
| PM | ASV96 | d__Bacteria | p__Firmicutes | c__Bacilli | o__Bacillales | f__Bacillaceae | g__Bacillus |
| PM | ASV93 | d__Bacteria | p__Firmicutes | c__Bacilli | o__Lactobacillales | f__Enterococcaceae | g__Enterococcus |
| PM | ASV92 | d__Bacteria | p__Gemmatimonadota | c__S0134_terrestrial_group | o__S0134_terrestrial_group | f__S0134_terrestrial_group | g__S0134_terrestrial_group |
| PM | ASV90 | d__Bacteria | p__Firmicutes | c__Bacilli | o__Bacillales | f__Marinococcaceae | g__Marinococcaceae |
| PM | ASV9 | d__Bacteria | p__Myxococcota | c__Myxococcia | o__Myxococcales | f__Vulgatibacteraceae | g__Vulgatibacter |
| PM | ASV82 | d__Bacteria | p__Firmicutes | c__Bacilli | o__Lactobacillales | f__Enterococcaceae | g__Enterococcus |
| PM | ASV75 | d__Bacteria | p__Firmicutes | c__Bacilli | o__Bacillales | f__Bacillaceae | g__Pseudogracilibacillus |
| PM | ASV71 | d__Bacteria | p__Acidobacteriota | c__Vicinamibacteria | o__Vicinamibacterales | f__uncultured | g__uncultured |
| PM | ASV70 | d__Bacteria | p__Firmicutes | c__Bacilli | o__Thermoactinomycetales | f__Thermoactinomycetaceae | g__uncultured |
| PM | ASV7 | d__Bacteria | p__Myxococcota | c__Myxococcia | o__Myxococcales | f__Vulgatibacteraceae | g__Vulgatibacter |
| PM | ASV69 | d__Bacteria | p__Firmicutes | c__Bacilli | o__Bacillales | f__Bacillaceae | g__Pseudogracilibacillus |
| PM | ASV53 | d__Bacteria | p__Bdellovibrionota | c__Bdellovibrionia | o__Bacteriovoracales | f__Bacteriovoracaceae | g__Peredibacter |
| PM | ASV48 | d__Bacteria | p__Firmicutes | c__Bacilli | o__Caldalkalibacillales | f__Caldalkalibacillaceae | g__Caldalkalibacillus |
| PM | ASV42 | d__Bacteria | p__Proteobacteria | c__Gammaproteobacteria | o__Pseudomonadales | f__Moraxellaceae | g__Acinetobacter |
| PM | ASV396 | d__Bacteria | p__Firmicutes | c__Bacilli | o__Bacillales | f__Bacillaceae | g__Pseudogracilibacillus |
| PM | ASV390 | d__Bacteria | p__Myxococcota | c__Myxococcia | o__Myxococcales | f__Vulgatibacteraceae | g__Vulgatibacter |
| PM | ASV387 | d__Bacteria | p__Gemmatimonadota | c__S0134_terrestrial_group | o__S0134_terrestrial_group | f__S0134_terrestrial_group | g__S0134_terrestrial_group |
| PM | ASV383 | d__Bacteria | p__Firmicutes | c__Bacilli | o__Bacillales | f__Bacillaceae | g__Cerasibacillus |
| PM | ASV381 | d__Bacteria | p__Firmicutes | c__Bacilli | o__Bacillales | f__Bacillaceae | g__Pseudogracilibacillus |
| PM | ASV377 | d__Bacteria | p__Firmicutes | c__Bacilli | o__Bacillales | f__Marinococcaceae | g__Marinococcaceae |
| PM | ASV369 | d__Bacteria | p__Firmicutes | c__Bacilli | o__Bacillales | f__Bacillaceae | g__Bacillus |
| PM | ASV367 | d__Bacteria | p__Gemmatimonadota | c__S0134_terrestrial_group | o__S0134_terrestrial_group | f__S0134_terrestrial_group | g__S0134_terrestrial_group |
| PM | ASV365 | d__Bacteria | p__Proteobacteria | c__Gammaproteobacteria | o__Burkholderiales | f__Alcaligenaceae | g__Pusillimonas |
| PM | ASV362 | d__Bacteria | p__Firmicutes | c__Bacilli | o__Bacillales | f__Bacillaceae |  |
| PM | ASV360 | d__Bacteria | p__Myxococcota | c__Myxococcia | o__Myxococcales | f__Vulgatibacteraceae | g__Vulgatibacter |
| PM | ASV36 | d__Bacteria | p__Firmicutes | c__Bacilli | o__Caldalkalibacillales | f__Caldalkalibacillaceae | g__Caldalkalibacillus |
| PM | ASV358 | d__Bacteria | p__Firmicutes | c__Bacilli | o__Bacillales | f__Bacillaceae |  |
| PM | ASV356 | d__Bacteria | p__Firmicutes | c__Bacilli | o__Bacillales | f__Bacillaceae | g__Sinibacillus |
| PM | ASV345 | d__Bacteria | p__Firmicutes | c__Bacilli | o__Bacillales | f__Bacillaceae | g__Amphibacillus |
| PM | ASV341 | d__Bacteria | p__Proteobacteria | c__Gammaproteobacteria | o__Xanthomonadales | f__Rhodanobacteraceae | g__Pseudofulvimonas |
| PM | ASV338 | d__Bacteria | p__Firmicutes | c__Bacilli | o__Thermoactinomycetales | f__Thermoactinomycetaceae | g__Kroppenstedtia |
| PM | ASV330 | d__Bacteria | p__Proteobacteria | c__Gammaproteobacteria | o__Pseudomonadales | f__Moraxellaceae | g__Acinetobacter |
| PM | ASV311 | d__Bacteria | p__Firmicutes | c__Bacilli | o__Bacillales | f__Bacillaceae | g__Sinibacillus |
| PM | ASV309 | d__Bacteria | p__Firmicutes | c__Bacilli | o__Bacillales | f__Bacillaceae | g__Gracilibacillus |
| PM | ASV308 | d__Bacteria | p__Bacteroidota | c__Bacteroidia | o__Cytophagales | f__MWH-CFBk5 | g__MWH-CFBk5 |
| PM | ASV307 | d__Bacteria | p__Firmicutes | c__Bacilli | o__Bacillales | f__Bacillaceae | g__Bacillus |
| PM | ASV302 | d__Bacteria | p__Proteobacteria | c__Gammaproteobacteria | o__Pseudomonadales | f__Pseudomonadaceae | g__Thiopseudomonas |
| PM | ASV301 | d__Bacteria | p__Firmicutes | c__Bacilli | o__Thermoactinomycetales | f__Thermoactinomycetaceae | g__uncultured |
| PM | ASV294 | d__Bacteria | p__Firmicutes | c__Bacilli | o__Bacillales | f__Bacillaceae | g__Bacillus |
| PM | ASV293 | d__Bacteria | p__Gemmatimonadota | c__S0134_terrestrial_group | o__S0134_terrestrial_group | f__S0134_terrestrial_group | g__S0134_terrestrial_group |
| PM | ASV292 | d__Bacteria | p__Firmicutes | c__Bacilli | o__Bacillales | f__Bacillaceae | g__Bacillus |
| PM | ASV289 | d__Bacteria | p__Firmicutes | c__Bacilli | o__Caldalkalibacillales | f__Caldalkalibacillaceae | g__Caldalkalibacillus |
| PM | ASV287 | d__Bacteria | p__Firmicutes | c__Bacilli | o__Bacillales | f__Bacillaceae | g__Pseudogracilibacillus |
| PM | ASV281 | d__Bacteria | p__Gemmatimonadota | c__S0134_terrestrial_group | o__S0134_terrestrial_group | f__S0134_terrestrial_group | g__S0134_terrestrial_group |
| PM | ASV276 | d__Bacteria | p__Proteobacteria | c__Gammaproteobacteria | o__Pseudomonadales | f__Moraxellaceae | g__Acinetobacter |
| PM | ASV270 | d__Bacteria | p__Firmicutes | c__Bacilli | o__Caldalkalibacillales | f__Caldalkalibacillaceae | g__Caldalkalibacillus |
| PM | ASV269 | d__Bacteria | p__Firmicutes | c__Bacilli | o__Thermoactinomycetales | f__Thermoactinomycetaceae | g__Planifilum |
| PM | ASV266 | d__Bacteria | p__Verrucomicrobiota | c__Verrucomicrobiae | o__Verrucomicrobiales | f__DEV007 | g__DEV007 |
| PM | ASV262 | d__Bacteria | p__Firmicutes | c__Bacilli | o__Bacillales | f__Bacillaceae |  |
| PM | ASV256 | d__Bacteria | p__Gemmatimonadota | c__Longimicrobia | o__Longimicrobiales | f__Longimicrobiaceae | g__Longimicrobiaceae |
| PM | ASV253 | d__Bacteria | p__Proteobacteria | c__Gammaproteobacteria | o__Cardiobacteriales | f__Wohlfahrtiimonadaceae | g__Ignatzschineria |
| PM | ASV252 | d__Bacteria | p__Gemmatimonadota | c__S0134_terrestrial_group | o__S0134_terrestrial_group | f__S0134_terrestrial_group | g__S0134_terrestrial_group |
| PM | ASV240 | d__Bacteria | p__Proteobacteria | c__Alphaproteobacteria | o__Kiloniellales | f__Fodinicurvataceae | g__uncultured |
| PM | ASV237 | d__Bacteria | p__Proteobacteria | c__Gammaproteobacteria | o__Pseudomonadales | f__Pseudomonadaceae | g__Thiopseudomonas |
| PM | ASV236 | d__Bacteria | p__Firmicutes | c__Bacilli | o__Bacillales | f__Bacillaceae | g__Cerasibacillus |
| PM | ASV231 | d__Bacteria | p__Firmicutes | c__Bacilli | o__Thermoactinomycetales | f__Thermoactinomycetaceae | g__Planifilum |
| PM | ASV229 | d__Bacteria | p__Firmicutes | c__Bacilli | o__Bacillales | f__Bacillaceae | g__Bacillus |
| PM | ASV225 | d__Bacteria | p__Firmicutes | c__Clostridia | o__Peptococcales | f__Peptococcaceae | g__uncultured |
| PM | ASV221 | d__Bacteria | p__Proteobacteria | c__Gammaproteobacteria | o__Pseudomonadales | f__Moraxellaceae | g__Acinetobacter |
| PM | ASV220 | d__Bacteria | p__Gemmatimonadota | c__S0134_terrestrial_group | o__S0134_terrestrial_group | f__S0134_terrestrial_group | g__S0134_terrestrial_group |
| PM | ASV216 | d__Bacteria | p__Firmicutes | c__Bacilli | o__Bacillales | f__Bacillaceae |  |
| PM | ASV215 | d__Bacteria | p__Proteobacteria | c__Gammaproteobacteria | o__Pseudomonadales | f__Moraxellaceae | g__Acinetobacter |
| PM | ASV214 | d__Bacteria | p__Firmicutes | c__Bacilli | o__Bacillales | f__Bacillaceae | g__Sinibacillus |
| PM | ASV212 | d__Bacteria | p__Firmicutes | c__Bacilli | o__Thermoactinomycetales | f__Thermoactinomycetaceae | g__Planifilum |
| PM | ASV21 | d__Bacteria | p__Myxococcota | c__Polyangia | o__Polyangiales | f__Sandaracinaceae | g__uncultured |
| PM | ASV207 | d__Bacteria | p__Firmicutes | c__Bacilli | o__Bacillales | f__Bacillaceae | g__Bacillus |
| PM | ASV200 | d__Bacteria | p__Firmicutes | c__Bacilli | o__Bacillales | f__Bacillaceae |  |
| PM | ASV2 | d__Bacteria | p__Firmicutes | c__Bacilli | o__Bacillales | f__Bacillaceae |  |
| PM | ASV197 | d__Bacteria | p__Myxococcota | c__Polyangia | o__Polyangiales | f__Sandaracinaceae | g__uncultured |
| PM | ASV193 | d__Bacteria | p__Firmicutes | c__Bacilli | o__Bacillales | f__Bacillaceae | g__Pseudogracilibacillus |
| PM | ASV191 | d__Bacteria | p__Proteobacteria | c__Gammaproteobacteria | o__Pseudomonadales | f__Moraxellaceae | g__Acinetobacter |
| PM | ASV188 | d__Bacteria | p__Proteobacteria | c__Gammaproteobacteria | o__Pseudomonadales | f__Pseudomonadaceae | g__Thiopseudomonas |
| PM | ASV187 | d__Bacteria | p__Proteobacteria | c__Gammaproteobacteria | o__Pseudomonadales | f__Moraxellaceae | g__Acinetobacter |
| PM | ASV186 | d__Bacteria | p__Firmicutes | c__Bacilli | o__Thermoactinomycetales | f__Thermoactinomycetaceae | g__Thermoactinomyces |
| PM | ASV178 | d__Bacteria | p__Gemmatimonadota | c__S0134_terrestrial_group | o__S0134_terrestrial_group | f__S0134_terrestrial_group | g__S0134_terrestrial_group |
| PM | ASV171 | d__Bacteria | p__Gemmatimonadota | c__S0134_terrestrial_group | o__S0134_terrestrial_group | f__S0134_terrestrial_group | g__S0134_terrestrial_group |
| PM | ASV166 | d__Bacteria | p__Firmicutes | c__Bacilli | o__Bacillales | f__Bacillaceae | g__Bacillus |
| PM | ASV164 | d__Bacteria | p__Firmicutes | c__Bacilli | o__Caldalkalibacillales | f__Caldalkalibacillaceae | g__Caldalkalibacillus |
| PM | ASV15 | d__Bacteria | p__Firmicutes | c__Bacilli | o__Bacillales | f__Bacillaceae | g__Sinibacillus |
| PM | ASV148 | d__Bacteria | p__Firmicutes | c__Bacilli | o__Bacillales | f__Bacillaceae | g__Sinibacillus |
| PM | ASV139 | d__Bacteria | p__Gemmatimonadota | c__Longimicrobia | o__Longimicrobiales | f__Longimicrobiaceae | g__Longimicrobiaceae |
| PM | ASV134 | d__Bacteria | p__Proteobacteria | c__Gammaproteobacteria | o__Pseudomonadales | f__Moraxellaceae | g__Acinetobacter |
| PM | ASV13 | d__Bacteria | p__Gemmatimonadota | c__S0134_terrestrial_group | o__S0134_terrestrial_group | f__S0134_terrestrial_group | g__S0134_terrestrial_group |
| PM | ASV129 | d__Bacteria | p__Myxococcota | c__Myxococcia | o__Myxococcales | f__Vulgatibacteraceae | g__Vulgatibacter |
| PM | ASV123 | d__Bacteria | p__Proteobacteria | c__Gammaproteobacteria | o__Pseudomonadales | f__Moraxellaceae | g__Acinetobacter |
| PM | ASV120 | d__Bacteria | p__Proteobacteria | c__Gammaproteobacteria | o__Pseudomonadales | f__Moraxellaceae | g__Acinetobacter |
| PM | ASV114 | d__Bacteria | p__Myxococcota | c__Myxococcia | o__Myxococcales | f__Vulgatibacteraceae | g__Vulgatibacter |
| PM | ASV111 | d__Bacteria | p__Firmicutes | c__Bacilli | o__Bacillales | f__Bacillaceae |  |
| PM | arsA | Miltidrug | ARGs | ARGs | ARGs | ARGs | ARGs |
| PM | aphA1 | Aminoglycoside | ARGs | ARGs | ARGs | ARGs | ARGs |
| PM | acrB | Miltidrug | ARGs | ARGs | ARGs | ARGs | ARGs |
| PM | acrA | Miltidrug | ARGs | ARGs | ARGs | ARGs | ARGs |
| PM | aac(6')-II | Aminoglycoside | ARGs | ARGs | ARGs | ARGs | ARGs |
| PM | aac(3)-xa | Aminoglycoside | ARGs | ARGs | ARGs | ARGs | ARGs |
| PMNG | vat(A) | MLSB | ARGs | ARGs | ARGs | ARGs | ARGs |
| PMNG | vanC | Vancomycin | ARGs | ARGs | ARGs | ARGs | ARGs |
| PMNG | vanA | Vancomycin | ARGs | ARGs | ARGs | ARGs | ARGs |
| PMNG | tra-A | MGEs | MGEs | MGEs | MGEs | MGEs | MGEs |
| PMNG | tetK | Tetracycline | ARGs | ARGs | ARGs | ARGs | ARGs |
| PMNG | qnrD | Miltidrug | ARGs | ARGs | ARGs | ARGs | ARGs |
| PMNG | mefA | MLSB | ARGs | ARGs | ARGs | ARGs | ARGs |
| PMNG | mdtA | Miltidrug | ARGs | ARGs | ARGs | ARGs | ARGs |
| PMNG | ISCR1 | MGEs | MGEs | MGEs | MGEs | MGEs | MGEs |
| PMNG | IS3 | MGEs | MGEs | MGEs | MGEs | MGEs | MGEs |
| PMNG | folA | Sulfonamide | ARGs | ARGs | ARGs | ARGs | ARGs |
| PMNG | ASV96 | d__Bacteria | p__Firmicutes | c__Bacilli | o__Bacillales | f__Bacillaceae | g__Bacillus |
| PMNG | ASV93 | d__Bacteria | p__Firmicutes | c__Bacilli | o__Lactobacillales | f__Enterococcaceae | g__Enterococcus |
| PMNG | ASV92 | d__Bacteria | p__Gemmatimonadota | c__S0134_terrestrial_group | o__S0134_terrestrial_group | f__S0134_terrestrial_group | g__S0134_terrestrial_group |
| PMNG | ASV90 | d__Bacteria | p__Firmicutes | c__Bacilli | o__Bacillales | f__Marinococcaceae | g__Marinococcaceae |
| PMNG | ASV9 | d__Bacteria | p__Myxococcota | c__Myxococcia | o__Myxococcales | f__Vulgatibacteraceae | g__Vulgatibacter |
| PMNG | ASV82 | d__Bacteria | p__Firmicutes | c__Bacilli | o__Lactobacillales | f__Enterococcaceae | g__Enterococcus |
| PMNG | ASV81 | d__Bacteria | p__Firmicutes | c__Bacilli | o__Thermoactinomycetales | f__Thermoactinomycetaceae | g__Novibacillus |
| PMNG | ASV75 | d__Bacteria | p__Firmicutes | c__Bacilli | o__Bacillales | f__Bacillaceae | g__Pseudogracilibacillus |
| PMNG | ASV73 | d__Bacteria | p__Proteobacteria | c__Gammaproteobacteria | o__Burkholderiales | f__Alcaligenaceae |  |
| PMNG | ASV70 | d__Bacteria | p__Firmicutes | c__Bacilli | o__Thermoactinomycetales | f__Thermoactinomycetaceae | g__uncultured |
| PMNG | ASV67 | d__Bacteria | p__Firmicutes | c__Bacilli | o__Bacillales | f__Bacillaceae |  |
| PMNG | ASV55 | d__Bacteria | p__Proteobacteria | c__Gammaproteobacteria | o__Pseudomonadales | f__Moraxellaceae | g__Acinetobacter |
| PMNG | ASV49 | d__Bacteria | p__Firmicutes | c__Bacilli | o__Bacillales | f__Bacillaceae |  |
| PMNG | ASV42 | d__Bacteria | p__Proteobacteria | c__Gammaproteobacteria | o__Pseudomonadales | f__Moraxellaceae | g__Acinetobacter |
| PMNG | ASV393 | d__Bacteria | p__Firmicutes | c__Bacilli | o__Bacillales | f__Bacillaceae | g__Bacillus |
| PMNG | ASV383 | d__Bacteria | p__Firmicutes | c__Bacilli | o__Bacillales | f__Bacillaceae | g__Cerasibacillus |
| PMNG | ASV381 | d__Bacteria | p__Firmicutes | c__Bacilli | o__Bacillales | f__Bacillaceae | g__Pseudogracilibacillus |
| PMNG | ASV38 | d__Bacteria | p__Proteobacteria | c__Gammaproteobacteria | o__Cardiobacteriales | f__Wohlfahrtiimonadaceae | g__Ignatzschineria |
| PMNG | ASV377 | d__Bacteria | p__Firmicutes | c__Bacilli | o__Bacillales | f__Marinococcaceae | g__Marinococcaceae |
| PMNG | ASV375 | d__Bacteria | p__Firmicutes | c__Bacilli | o__Lactobacillales | f__Enterococcaceae | g__Enterococcus |
| PMNG | ASV369 | d__Bacteria | p__Firmicutes | c__Bacilli | o__Bacillales | f__Bacillaceae | g__Bacillus |
| PMNG | ASV367 | d__Bacteria | p__Gemmatimonadota | c__S0134_terrestrial_group | o__S0134_terrestrial_group | f__S0134_terrestrial_group | g__S0134_terrestrial_group |
| PMNG | ASV360 | d__Bacteria | p__Myxococcota | c__Myxococcia | o__Myxococcales | f__Vulgatibacteraceae | g__Vulgatibacter |
| PMNG | ASV358 | d__Bacteria | p__Firmicutes | c__Bacilli | o__Bacillales | f__Bacillaceae |  |
| PMNG | ASV356 | d__Bacteria | p__Firmicutes | c__Bacilli | o__Bacillales | f__Bacillaceae | g__Sinibacillus |
| PMNG | ASV353 | d__Bacteria | p__Myxococcota | c__Polyangia | o__Polyangiales | f__Sandaracinaceae | g__uncultured |
| PMNG | ASV352 | d__Bacteria | p__Firmicutes | c__Bacilli | o__Bacillales | f__Bacillaceae | g__Bacillus |
| PMNG | ASV35 | d__Bacteria | p__Proteobacteria | c__Gammaproteobacteria | o__Pseudomonadales | f__Pseudomonadaceae | g__Oblitimonas |
| PMNG | ASV348 | d__Bacteria | p__Proteobacteria | c__Gammaproteobacteria | o__Cardiobacteriales | f__Wohlfahrtiimonadaceae | g__Ignatzschineria |
| PMNG | ASV335 | d__Bacteria | p__Proteobacteria | c__Gammaproteobacteria | o__Burkholderiales | f__Alcaligenaceae | g__Pusillimonas |
| PMNG | ASV325 | d__Bacteria | p__Proteobacteria | c__Gammaproteobacteria | o__Pseudomonadales | f__Moraxellaceae | g__Acinetobacter |
| PMNG | ASV322 | d__Bacteria | p__Firmicutes | c__Bacilli | o__Bacillales | f__Bacillaceae |  |
| PMNG | ASV320 | d__Bacteria | p__Firmicutes | c__Bacilli | o__Lactobacillales | f__Enterococcaceae |  |
| PMNG | ASV311 | d__Bacteria | p__Firmicutes | c__Bacilli | o__Bacillales | f__Bacillaceae | g__Sinibacillus |
| PMNG | ASV309 | d__Bacteria | p__Firmicutes | c__Bacilli | o__Bacillales | f__Bacillaceae | g__Gracilibacillus |
| PMNG | ASV307 | d__Bacteria | p__Firmicutes | c__Bacilli | o__Bacillales | f__Bacillaceae | g__Bacillus |
| PMNG | ASV306 | d__Bacteria | p__Proteobacteria | c__Gammaproteobacteria | o__Cardiobacteriales | f__Wohlfahrtiimonadaceae | g__Ignatzschineria |
| PMNG | ASV293 | d__Bacteria | p__Gemmatimonadota | c__S0134_terrestrial_group | o__S0134_terrestrial_group | f__S0134_terrestrial_group | g__S0134_terrestrial_group |
| PMNG | ASV292 | d__Bacteria | p__Firmicutes | c__Bacilli | o__Bacillales | f__Bacillaceae | g__Bacillus |
| PMNG | ASV283 | d__Bacteria | p__Firmicutes | c__Bacilli | o__Bacillales | f__Bacillaceae | g__Bacillus |
| PMNG | ASV277 | d__Bacteria | p__Gemmatimonadota | c__S0134_terrestrial_group | o__S0134_terrestrial_group | f__S0134_terrestrial_group | g__S0134_terrestrial_group |
| PMNG | ASV276 | d__Bacteria | p__Proteobacteria | c__Gammaproteobacteria | o__Pseudomonadales | f__Moraxellaceae | g__Acinetobacter |
| PMNG | ASV270 | d__Bacteria | p__Firmicutes | c__Bacilli | o__Caldalkalibacillales | f__Caldalkalibacillaceae | g__Caldalkalibacillus |
| PMNG | ASV269 | d__Bacteria | p__Firmicutes | c__Bacilli | o__Thermoactinomycetales | f__Thermoactinomycetaceae | g__Planifilum |
| PMNG | ASV266 | d__Bacteria | p__Verrucomicrobiota | c__Verrucomicrobiae | o__Verrucomicrobiales | f__DEV007 | g__DEV007 |
| PMNG | ASV262 | d__Bacteria | p__Firmicutes | c__Bacilli | o__Bacillales | f__Bacillaceae |  |
| PMNG | ASV255 | d__Bacteria | p__Firmicutes | c__Bacilli | o__Bacillales | f__Bacillaceae |  |
| PMNG | ASV253 | d__Bacteria | p__Proteobacteria | c__Gammaproteobacteria | o__Cardiobacteriales | f__Wohlfahrtiimonadaceae | g__Ignatzschineria |
| PMNG | ASV231 | d__Bacteria | p__Firmicutes | c__Bacilli | o__Thermoactinomycetales | f__Thermoactinomycetaceae | g__Planifilum |
| PMNG | ASV221 | d__Bacteria | p__Proteobacteria | c__Gammaproteobacteria | o__Pseudomonadales | f__Moraxellaceae | g__Acinetobacter |
| PMNG | ASV215 | d__Bacteria | p__Proteobacteria | c__Gammaproteobacteria | o__Pseudomonadales | f__Moraxellaceae | g__Acinetobacter |
| PMNG | ASV213 | d__Bacteria | p__Firmicutes | c__Bacilli | o__Bacillales | f__Bacillaceae | g__Pseudogracilibacillus |
| PMNG | ASV21 | d__Bacteria | p__Myxococcota | c__Polyangia | o__Polyangiales | f__Sandaracinaceae | g__uncultured |
| PMNG | ASV207 | d__Bacteria | p__Firmicutes | c__Bacilli | o__Bacillales | f__Bacillaceae | g__Bacillus |
| PMNG | ASV205 | d__Bacteria | p__Firmicutes | c__Bacilli | o__Bacillales | f__Bacillaceae | g__Bacillus |
| PMNG | ASV2 | d__Bacteria | p__Firmicutes | c__Bacilli | o__Bacillales | f__Bacillaceae |  |
| PMNG | ASV197 | d__Bacteria | p__Myxococcota | c__Polyangia | o__Polyangiales | f__Sandaracinaceae | g__uncultured |
| PMNG | ASV193 | d__Bacteria | p__Firmicutes | c__Bacilli | o__Bacillales | f__Bacillaceae | g__Pseudogracilibacillus |
| PMNG | ASV191 | d__Bacteria | p__Proteobacteria | c__Gammaproteobacteria | o__Pseudomonadales | f__Moraxellaceae | g__Acinetobacter |
| PMNG | ASV188 | d__Bacteria | p__Proteobacteria | c__Gammaproteobacteria | o__Pseudomonadales | f__Pseudomonadaceae | g__Thiopseudomonas |
| PMNG | ASV186 | d__Bacteria | p__Firmicutes | c__Bacilli | o__Thermoactinomycetales | f__Thermoactinomycetaceae | g__Thermoactinomyces |
| PMNG | ASV178 | d__Bacteria | p__Gemmatimonadota | c__S0134_terrestrial_group | o__S0134_terrestrial_group | f__S0134_terrestrial_group | g__S0134_terrestrial_group |
| PMNG | ASV171 | d__Bacteria | p__Gemmatimonadota | c__S0134_terrestrial_group | o__S0134_terrestrial_group | f__S0134_terrestrial_group | g__S0134_terrestrial_group |
| PMNG | ASV169 | d__Bacteria | p__Proteobacteria | c__Gammaproteobacteria | o__Pseudomonadales | f__Moraxellaceae | g__Acinetobacter |
| PMNG | ASV166 | d__Bacteria | p__Firmicutes | c__Bacilli | o__Bacillales | f__Bacillaceae | g__Bacillus |
| PMNG | ASV151 | d__Bacteria | p__Proteobacteria | c__Gammaproteobacteria | o__Cardiobacteriales | f__Wohlfahrtiimonadaceae | g__Ignatzschineria |
| PMNG | ASV15 | d__Bacteria | p__Firmicutes | c__Bacilli | o__Bacillales | f__Bacillaceae | g__Sinibacillus |
| PMNG | ASV148 | d__Bacteria | p__Firmicutes | c__Bacilli | o__Bacillales | f__Bacillaceae | g__Sinibacillus |
| PMNG | ASV146 | d__Bacteria | p__Firmicutes | c__Bacilli | o__Bacillales | f__Bacillaceae |  |
| PMNG | ASV141 | d__Bacteria | p__Firmicutes | c__Bacilli | o__Bacillales | f__Bacillaceae | g__Bacillus |
| PMNG | ASV139 | d__Bacteria | p__Gemmatimonadota | c__Longimicrobia | o__Longimicrobiales | f__Longimicrobiaceae | g__Longimicrobiaceae |
| PMNG | ASV13 | d__Bacteria | p__Gemmatimonadota | c__S0134_terrestrial_group | o__S0134_terrestrial_group | f__S0134_terrestrial_group | g__S0134_terrestrial_group |
| PMNG | ASV120 | d__Bacteria | p__Proteobacteria | c__Gammaproteobacteria | o__Pseudomonadales | f__Moraxellaceae | g__Acinetobacter |
| PMNG | ASV112 | d__Bacteria | p__Firmicutes | c__Bacilli | o__Bacillales | f__Bacillaceae | g__uncultured |
| PMNG | ASV111 | d__Bacteria | p__Firmicutes | c__Bacilli | o__Bacillales | f__Bacillaceae |  |
| PMNG | arsA | Miltidrug | ARGs | ARGs | ARGs | ARGs | ARGs |
| PMNG | acrB | Miltidrug | ARGs | ARGs | ARGs | ARGs | ARGs |
| PMNG | aac(6')-II | Aminoglycoside | ARGs | ARGs | ARGs | ARGs | ARGs |
| PMCH | vat(A) | MLSB | ARGs | ARGs | ARGs | ARGs | ARGs |
| PMCH | vanC | Vancomycin | ARGs | ARGs | ARGs | ARGs | ARGs |
| PMCH | tnpA-1 | MGEs | MGEs | MGEs | MGEs | MGEs | MGEs |
| PMCH | tetW | Tetracycline | ARGs | ARGs | ARGs | ARGs | ARGs |
| PMCH | tetT | Tetracycline | ARGs | ARGs | ARGs | ARGs | ARGs |
| PMCH | tetR | Tetracycline | ARGs | ARGs | ARGs | ARGs | ARGs |
| PMCH | tetO | Tetracycline | ARGs | ARGs | ARGs | ARGs | ARGs |
| PMCH | tetC | Tetracycline | ARGs | ARGs | ARGs | ARGs | ARGs |
| PMCH | tetA | Tetracycline | ARGs | ARGs | ARGs | ARGs | ARGs |
| PMCH | sul1 | Sulfonamide | ARGs | ARGs | ARGs | ARGs | ARGs |
| PMCH | qnrS2 | Miltidrug | ARGs | ARGs | ARGs | ARGs | ARGs |
| PMCH | msr(A) | MLSB | ARGs | ARGs | ARGs | ARGs | ARGs |
| PMCH | mefA | MLSB | ARGs | ARGs | ARGs | ARGs | ARGs |
| PMCH | lnuA | MLSB | ARGs | ARGs | ARGs | ARGs | ARGs |
| PMCH | ISCR1 | MGEs | MGEs | MGEs | MGEs | MGEs | MGEs |
| PMCH | IS3 | MGEs | MGEs | MGEs | MGEs | MGEs | MGEs |
| PMCH | IS26 | MGEs | MGEs | MGEs | MGEs | MGEs | MGEs |
| PMCH | intl3 | MGEs | MGEs | MGEs | MGEs | MGEs | MGEs |
| PMCH | intl2 | MGEs | MGEs | MGEs | MGEs | MGEs | MGEs |
| PMCH | folA | Sulfonamide | ARGs | ARGs | ARGs | ARGs | ARGs |
| PMCH | dfrC | Trimethoprim | ARGs | ARGs | ARGs | ARGs | ARGs |
| PMCH | dfrA1 | Sulfonamide | ARGs | ARGs | ARGs | ARGs | ARGs |
| PMCH | ASV93 | d__Bacteria | p__Firmicutes | c__Bacilli | o__Lactobacillales | f__Enterococcaceae | g__Enterococcus |
| PMCH | ASV82 | d__Bacteria | p__Firmicutes | c__Bacilli | o__Lactobacillales | f__Enterococcaceae | g__Enterococcus |
| PMCH | ASV75 | d__Bacteria | p__Firmicutes | c__Bacilli | o__Bacillales | f__Bacillaceae | g__Pseudogracilibacillus |
| PMCH | ASV71 | d__Bacteria | p__Acidobacteriota | c__Vicinamibacteria | o__Vicinamibacterales | f__uncultured | g__uncultured |
| PMCH | ASV69 | d__Bacteria | p__Firmicutes | c__Bacilli | o__Bacillales | f__Bacillaceae | g__Pseudogracilibacillus |
| PMCH | ASV56 | d__Bacteria | p__Proteobacteria | c__Gammaproteobacteria | o__Cardiobacteriales | f__Wohlfahrtiimonadaceae | g__Wohlfahrtiimonas |
| PMCH | ASV47 | d__Bacteria | p__Firmicutes | c__Bacilli | o__Bacillales | f__Bacillaceae | g__Pseudogracilibacillus |
| PMCH | ASV381 | d__Bacteria | p__Firmicutes | c__Bacilli | o__Bacillales | f__Bacillaceae | g__Pseudogracilibacillus |
| PMCH | ASV375 | d__Bacteria | p__Firmicutes | c__Bacilli | o__Lactobacillales | f__Enterococcaceae | g__Enterococcus |
| PMCH | ASV360 | d__Bacteria | p__Myxococcota | c__Myxococcia | o__Myxococcales | f__Vulgatibacteraceae | g__Vulgatibacter |
| PMCH | ASV359 | d__Bacteria | p__Proteobacteria | c__Gammaproteobacteria | o__Pseudomonadales | f__Moraxellaceae | g__Acinetobacter |
| PMCH | ASV356 | d__Bacteria | p__Firmicutes | c__Bacilli | o__Bacillales | f__Bacillaceae | g__Sinibacillus |
| PMCH | ASV320 | d__Bacteria | p__Firmicutes | c__Bacilli | o__Lactobacillales | f__Enterococcaceae |  |
| PMCH | ASV32 | d__Bacteria | p__Desulfobacterota | c__Desulfobulbia | o__Desulfobulbales | f__Desulfobulbaceae | g__Desulfobulbus |
| PMCH | ASV311 | d__Bacteria | p__Firmicutes | c__Bacilli | o__Bacillales | f__Bacillaceae | g__Sinibacillus |
| PMCH | ASV309 | d__Bacteria | p__Firmicutes | c__Bacilli | o__Bacillales | f__Bacillaceae | g__Gracilibacillus |
| PMCH | ASV293 | d__Bacteria | p__Gemmatimonadota | c__S0134_terrestrial_group | o__S0134_terrestrial_group | f__S0134_terrestrial_group | g__S0134_terrestrial_group |
| PMCH | ASV292 | d__Bacteria | p__Firmicutes | c__Bacilli | o__Bacillales | f__Bacillaceae | g__Bacillus |
| PMCH | ASV248 | d__Bacteria | p__Firmicutes | c__Bacilli | o__Bacillales | f__Bacillaceae | g__Cerasibacillus |
| PMCH | ASV234 | d__Bacteria | p__Firmicutes | c__Bacilli | o__Lactobacillales | f__Enterococcaceae | g__Enterococcus |
| PMCH | ASV215 | d__Bacteria | p__Proteobacteria | c__Gammaproteobacteria | o__Pseudomonadales | f__Moraxellaceae | g__Acinetobacter |
| PMCH | ASV214 | d__Bacteria | p__Firmicutes | c__Bacilli | o__Bacillales | f__Bacillaceae | g__Sinibacillus |
| PMCH | ASV21 | d__Bacteria | p__Myxococcota | c__Polyangia | o__Polyangiales | f__Sandaracinaceae | g__uncultured |
| PMCH | ASV2 | d__Bacteria | p__Firmicutes | c__Bacilli | o__Bacillales | f__Bacillaceae |  |
| PMCH | ASV193 | d__Bacteria | p__Firmicutes | c__Bacilli | o__Bacillales | f__Bacillaceae | g__Pseudogracilibacillus |
| PMCH | ASV173 | d__Bacteria | p__Proteobacteria | c__Gammaproteobacteria | o__Pseudomonadales | f__Moraxellaceae | g__Acinetobacter |
| PMCH | ASV171 | d__Bacteria | p__Gemmatimonadota | c__S0134_terrestrial_group | o__S0134_terrestrial_group | f__S0134_terrestrial_group | g__S0134_terrestrial_group |
| PMCH | ASV151 | d__Bacteria | p__Proteobacteria | c__Gammaproteobacteria | o__Cardiobacteriales | f__Wohlfahrtiimonadaceae | g__Ignatzschineria |
| PMCH | ASV140 | d__Bacteria | p__Firmicutes | c__Bacilli | o__Bacillales | f__Bacillaceae | g__Pseudogracilibacillus |
| PMCH | ASV139 | d__Bacteria | p__Gemmatimonadota | c__Longimicrobia | o__Longimicrobiales | f__Longimicrobiaceae | g__Longimicrobiaceae |
| PMCH | ASV138 | d__Bacteria | p__Desulfobacterota | c__Desulfovibrionia | o__Desulfovibrionales | f__Desulfovibrionaceae | g__uncultured |
| PMCH | ASV120 | d__Bacteria | p__Proteobacteria | c__Gammaproteobacteria | o__Pseudomonadales | f__Moraxellaceae | g__Acinetobacter |
| PMCH | ASV106 | d__Bacteria | p__Proteobacteria | c__Gammaproteobacteria | o__Cardiobacteriales | f__Wohlfahrtiimonadaceae | g__Ignatzschineria |
| PMCH | aphA1 | Aminoglycoside | ARGs | ARGs | ARGs | ARGs | ARGs |
| PMCH | acrB | Miltidrug | ARGs | ARGs | ARGs | ARGs | ARGs |
| PMCH | acrA | Miltidrug | ARGs | ARGs | ARGs | ARGs | ARGs |
| PMCH | aadE | Aminoglycoside | ARGs | ARGs | ARGs | ARGs | ARGs |
| PMCH | aadD | Aminoglycoside | ARGs | ARGs | ARGs | ARGs | ARGs |
| PMCH | aacA/aphD | Aminoglycoside | ARGs | ARGs | ARGs | ARGs | ARGs |
| PMCH | Aac6-Aph2 | Aminoglycoside | ARGs | ARGs | ARGs | ARGs | ARGs |


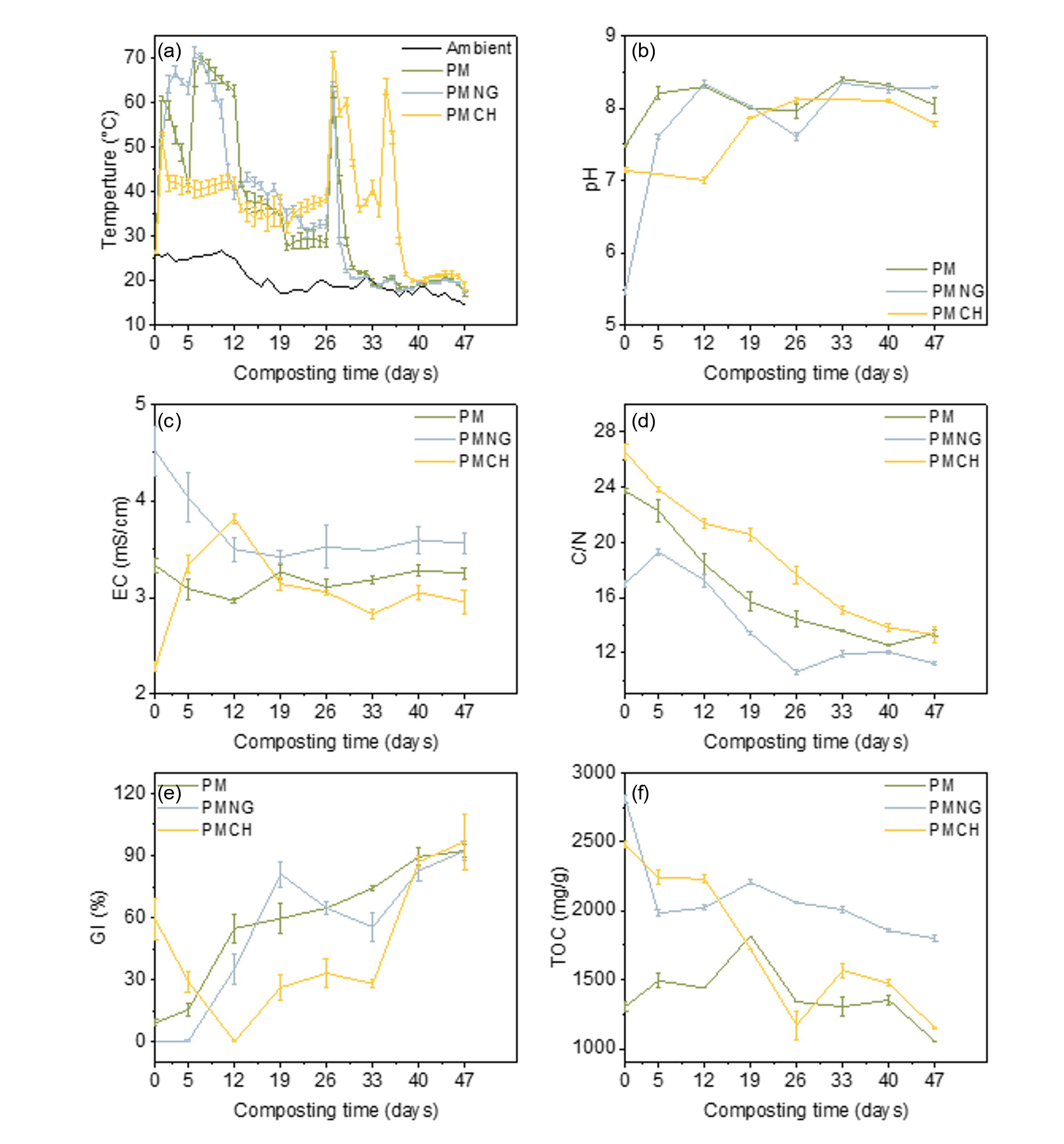


**Fig. S1.** Changes of (a) temperature, (b) pH, (c) EC, (d) total carbon to total nitrogen ratio (C/N), (e) germination index (GI), (f) total organic carbon (TOC) in different treatments during composting.


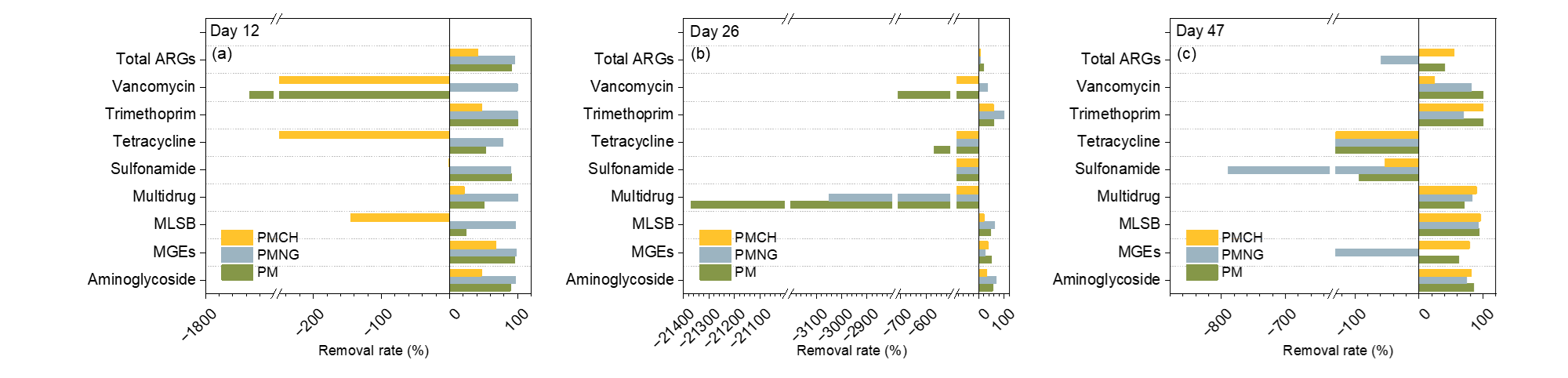


**Fig. S2.** The removal rate of ARGs and MGEs in thermophilic (a), cooling(b) and maturation(c) periods relative to day 0 in different treatments during composting.
